# Editorial: DNA damage response in the context of chromatin

**DOI:** 10.3389/fcell.2022.1095652

**Published:** 2023-01-10

**Authors:** Dali Zong, Burkhard Jakob, Lovisa Lundholm

**Affiliations:** ^1^ Laboratory of Genome Integrity, National Cancer Institute, National Institutes of Health, Bethesda, MD, United States; ^2^ Department of Biophysics, GSI Helmholtzzentrum für Schwerionenforschung GmbH, Darmstadt, Germany; ^3^ Department of Biology, Technische Universität Darmstadt, Darmstadt, Germany; ^4^ Department of Molecular Biosciences, The Wenner-Gren Institute, Stockholm University, Stockholm, Sweden

**Keywords:** DNA damage, DNA repair, chromatin, double strand break, post-translational modifications, pathway choice, clustered damage

All DNA transactions, including the repair of damaged DNA, take place in the context of a highly organized yet dynamic chromatin. While studies on individual proteins have yielded important insights into the constituent components of DNA repair machineries that evolved in various organisms, it has become abundantly clear that a more complete understanding of the molecular choreography of DNA repair must take into consideration the complex interplay between repair factors and the chromatin milieu in which they operate. This Research Topic collection, termed “DNA damage response in the context of chromatin”, brings together experts at the forefront of this emerging field with a series of authoritative reviews and exciting original articles to provide a timely update on our current understandings of biology at the intersection between chromatin and DNA repair.

Local chromatin landscapes can vary profoundly within a nucleus, which may significantly influence not only the initial generation of DNA damage, but also the subsequent activation of cellular DNA damage response (DDR) pathways (Chen and Sleckman). For instance, the compact silent heterochromatin (Chansard et al.) and the transcriptionally highly active ribosomal DNA clusters confined within the nucleolus would be expected to present different physical challenges for damage detection as well as repair. In parallel, the structure of DNA lesions and their proximity to one another can pose another set of challenges. In particular, clustered damage, as produced by heavy ion radiotherapy (Danforth et al.), or enzymatically induced (Mladenova et al.), is notoriously difficult to repair and may even recruit multiple competing or mutually antagonistic repair machineries. Recent studies have identified post-translational modifications of histones, especially ubiquitylation (Kolobynina et al.), as being essential for the activation of a productive DDR that couples correct repair pathway choice (Chen and Tyler) with maintenance of epigenome integrity. In addition, certain proteins have evolved to play pivotal roles in promoting specialized DDR processes, as exemplified by Treacle, a key regulator of the nucleolar DDR (Gal et al.) that is recurrently dysregulated in human cancers (Oxe and Larsen). Finally, the global spatial architecture of chromatin (e.g., chromatin loops or chromosome territories) also influences the DDR, significantly impacting on cellular fate (Zagelbaum and Gautier, 2022).

DNA damage incurred by the human body is inherently harmful. The bulk of these lesions originate endogenously, often in the form of oxidative base damages caused by stray metabolites and polymerase errors that occasionally occur during normal DNA replication. Others are produced by acute or prolonged exposure to external sources of genotoxins, including many environmental agents (e.g., ultraviolet rays, tobacco smoke, radon, heavy metal contaminants) and a host of chemo-radiotherapeutics. Nevertheless, it should be pointed out that certain types of DNA lesions can actually be generated as part of important physiological processes, including antigen receptor maturation in immune cells and meiosis in gametes (Khan and Ali, 2017). Moreover, certain developmental processes, such as those found in differentiating neurons, proceed through chromatin-directed DNA damage as an intermediate step (Wu et al., 2021; Wang et al., 2022). Unsurprisingly, mutations in chromatin regulators of DDR, whether inherited or somatically acquired, have been linked to a growing number of human ailments including aging. Thus, aside from providing vital clues about the basic biology of DNA repair, further elucidation of the chromatin response to DNA damage has important implications for human health and disease.

The past decade has witnessed an explosion of explorations in DNA repair in the context of chromatin, driven in part by rapid technological advances. Methods such as ChIP-seq, automated fluorescence imaging, CRISPR screens and proteomics are now routinely employed by researchers to probe DNA-chromatin interactions, and new approaches are continuously being developed. As guest editors of this Research Topic collection, we hope that the articles presented herein will be a valuable resource for the community and help inspire future experimental innovations.
